# Orbital pseudotumor in pediatrics: A single tertiary center experience

**DOI:** 10.1002/ccr3.7125

**Published:** 2023-03-17

**Authors:** Mohammed Almuqbil

**Affiliations:** ^1^ College of Medicine King Saud bin Abdulaziz University for Health Sciences (KSAU‐HS) Riyadh Saudi Arabia; ^2^ Division of Pediatric Neurology King Abdullah Specialist Children's Hospital (KASCH), National Guard Health Affairs (NGHA) Riyadh Saudi Arabia; ^3^ King Abdullah International Medical Research Center (KAIMRC), Ministry of National Guard Riyadh Saudi Arabia

**Keywords:** computed topography, magnetic resonance imaging, orbital pseudotumor

## Abstract

Orbital pseudotumor is a rare condition characterized by an idiopathic inflammatory process of the orbit with a polymorphous lymphoid infiltrate. It is misdiagnosed as orbital cellulitis or orbital mass with conjunctivitis in children.

## 1 INTRODUCTION

Orbital pseudotumor is a rare condition characterized by an idiopathic inflammatory process of the orbit with a polymorphous lymphoid infiltrate. It is misdiagnosed as orbital cellulitis or orbital mass with conjunctivitis in children. This study presents two pseudotumor cases among two boys presented with intermittent headache.

Orbital pseudotumor is a clinical condition that is an idiopathic inflammatory process of the orbit that is marked by an infiltrate of different kinds of lymphocytes.^1^, ^2^ The clinical characteristic of an orbital pseudotumor is that it is a benign, noninfectious, and space‐occupying lesion. Clinical presentation usually occurs in the 4th to 5th decades with signs and symptoms of proptosis, palpable mass, eyelid swelling, and visual problems.^3^ Orbital pseudotumors account for 6% to 16% of all orbital lesions and are a common entity requiring an orbital biopsy.^4^ Inflammatory pseudotumors are acute reactions that involve specific topographical sites such as the Tenon's space, perioptic nerve connective tissues, lacrimal glands, and extraocular muscles. Furthermore, hypocellular mixed inflammatory infiltrates with initial edema, followed by progressive fibrosis, are also one of the cardinal features. In addition, orbital pseudotumor also affects the orbital fat and forms an irregularly shaped infiltrative mass lesion due to fibrosis, which leads to motility restriction and optic nerve compression.^5^ The definitive diagnosis encompasses imaging, biopsy, fine needle aspiration, and histopathology.^6^ Here, we report a case series of two children diagnosed with orbital pseudotumor and its management.

## 2 RESULTS

### 2.1 Case 1

**FIGURE 1:**
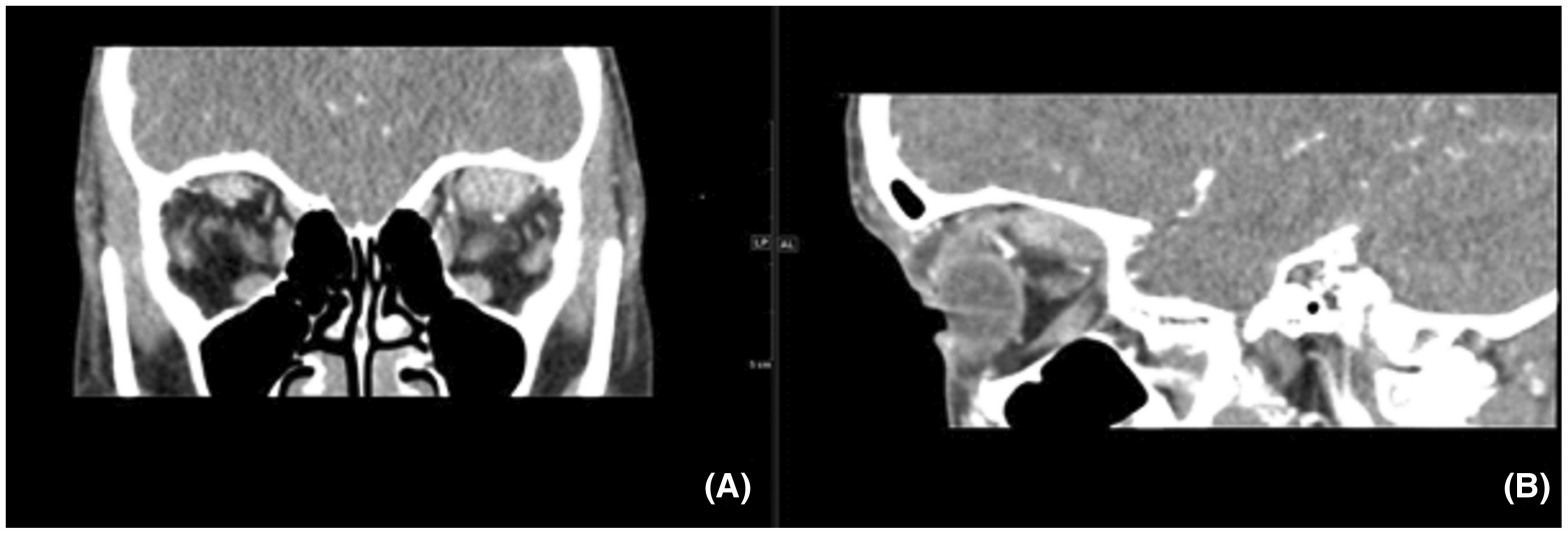
Contrast‐enhanced CT scan of the orbit reformatted images on coronal (A) and sagittal (B) images show diffusely enlarged left superior rectus muscle with intense homogenous enhancement with enhancement of its tendinous insertion.

A 13‐year‐old healthy boy, presented with a one‐week history of left upper eyelid swelling. His ophthalmological examination, apart from eyelid swelling causing mechanical ptosis, was normal. His eyelid swelling was switching from left to right. He had been complaining of intermittent migraine headaches for a year and had received no treatment. All routine blood investigations were within normal limits. The contrast‐enhanced CT scan of the patient is shown in Figure 1.

### 2.2 Case 2

**FIGURE 2:**
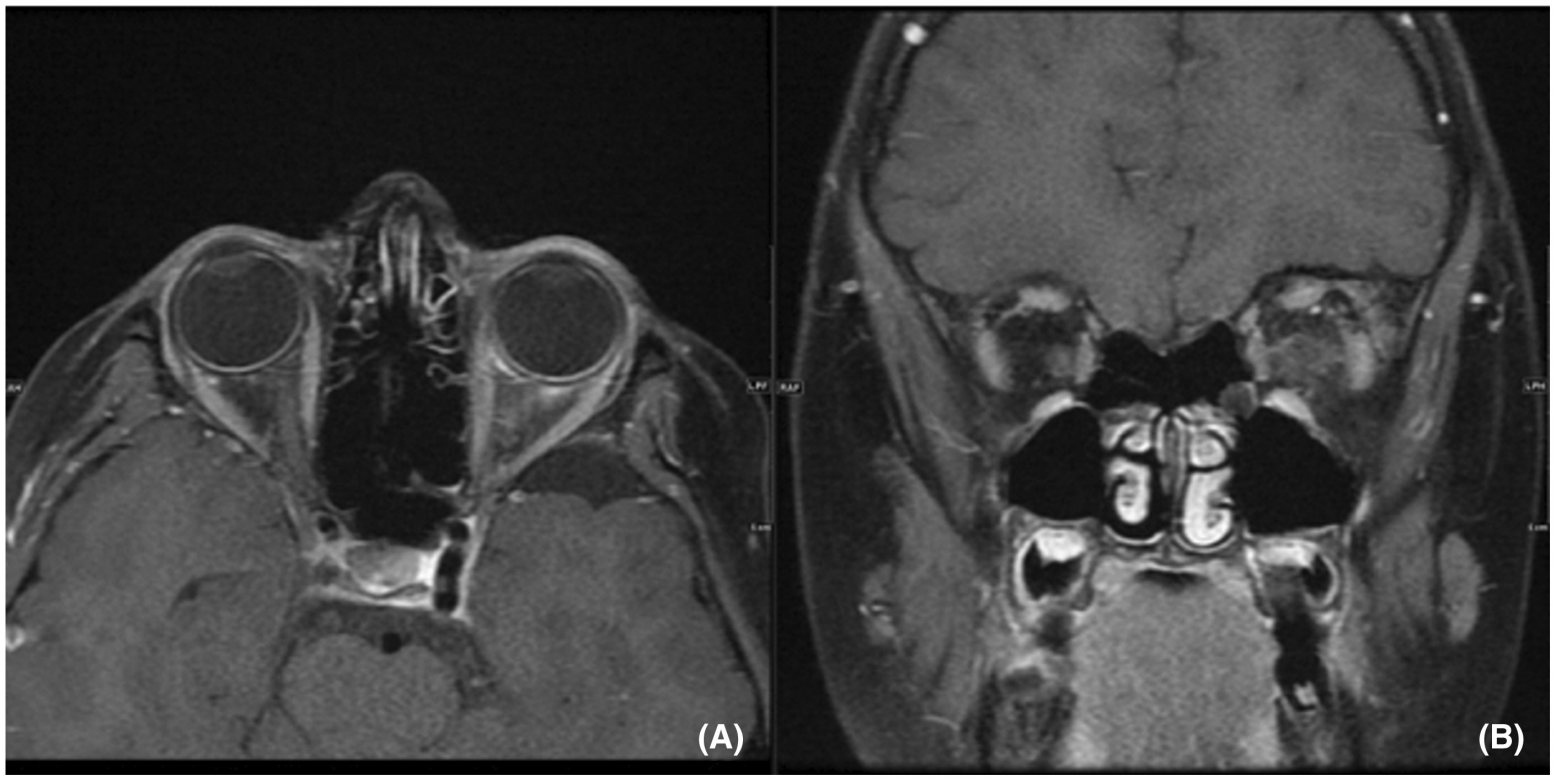
Contrast‐enhanced MR images of the orbit axial (A) and coronal (B) images show diffuse enhancement of the left lateral rectus as well as its tendinous insertion. Mild adjacent stranding of the orbit fat noted in keeping with associated cellulitis.

A nine‐year‐old healthy boy with a history of intermittent headache for 2 years presented to our emergency department with a one‐week history of increased pressure intensity (9/10), feeling retro‐orbital pressure, and left eye droopiness. On follow‐up, he was found to have left eye episcleritis. His headache was primarily left temporal, but shifting in location, and was associated with photophobia and phonophobia. It was pulsatile and was relieved by sleeping and acetaminophen. His ophthalmological and neurological examinations were normal. In the second case, investigations included routine lumbar puncture with opening pressure, lactic acid, ammonia, amino acids, complements, thyroid antibodies, erythrocyte sedimentation rate, myelin oligodendrocyte glycoprotein, aquaporin antibodies, oligoclonal bands, the IgG index, HLA B51, von Willebrand factor, and NMDA receptor antibodies, all within normal limits. Riboflavin 200 mg taken orally once daily controls his headache very well. Contrast‐enhanced axial MR images of the patient are shown in Figure 2.

## 3 DISCUSSION

Orbital pseudotumor was first highlighted by Birch Hirschfield in 1905. Although in most cases it is idiopathic, reports suggest that unknown microbes, minor injuries, and long‐term irritation are the major risk factors for the progression of the condition.^7^ Orbital pseudotumor is the third most common orbital disease after thyroid orbitopathy and lymphoproliferative disorder, and it encompasses approximately 10% of all orbital mass lesions.^8^ It can affect both sexes and people of any age or ethnicity, but is rare in childhood.^4^, ^5^ When compared to adults, the orbital pseudotumor in children usually has a bilateral pattern and has constitutional signs and symptoms, such as headache, fever, malaise, emesis, anorexia, lethargy, abdominal pain, and weight loss.

Palpable mass, decreased ocular motility, eyelid swelling, pain, proptosis, and increased intraocular pressure are all common ophthalmic clinical findings.^9^ Furthermore, iritis, eosinophilia, and optic disc edema are more commonly associated with children.^9^ Mimics of orbital pseudotumors include congenital orbital mass lesions or orbital neoplastic diseases such as lymphoma or rhabdomyosarcoma. Orbital inflammation can involve any of the orbital soft tissues, with the rectus muscles (myositis) and also orbital cellulitis, and the lacrimal gland (dacryoadenitis) as the most commonly involved sites.^2^

In the present case series, a contrast‐enhanced CT scan displays a diffusely enlarged left superior rectus muscle with intense homogenous enhancement and enhancement of its tendineous insertion (Figure 1), which indicates an orbital myositis. Orbital myositis is a common component of orbital pseudotumor, and 90–95% of cases are unilateral. Orbital myositis is considered a subgroup of orbital pseudotumors primarily involving the extraocular muscles. The classic appearance of orbital myositis includes a unilateral thickening of one or two extraocular muscles, often also involving the surrounding fat, tendon, and myotendinous junction. The most frequently affected muscle is the inferior rectus; although we report a case involving the superior rectus muscle. Echography and a CT scan reveal enlarged muscle bellies and thickened tendons with low internal reflectivity.^10^ Although orbital myositis is generally considered to be an idiopathic inflammation, in certain patients it may be a manifestation of Lyme disease.^10^

In the present case series, another child presented with episcleritis and retro‐orbital pressure. In cases of orbital pseudotumor, inflammatory cellular infiltrates have been demonstrated within involved muscles, sclera, and episclera, as well as in the intraorbital connective tissue, forming focal tissue masses. Myositic, tumefactive, episcleritic, and infiltrative (diffuse) forms have been described both alone and in combination. Isolated scleral inflammation, as seen in our patients, characterized by thickening and enhancement of the sclerouveal rim, probably represents a type of orbital pseudotumor.^11^

Bernardino et al., reviewed 350 normal patients and patients with orbital pseudotumor.^12^ In the normal patients, the sclerouveal rim was 3.2–4 mm thick; whereas, in the patients with pseudotumors, the sclerouveal rim was 1.5–3.5 times thicker than normal. In three of eight patients with sclerouveal rim thickening and orbital pseudotumors studied by Bernardino et al., the scleral changes were the predominant feature. CT can easily assess the other orbital structures involved by an orbital pseudotumor.

Sclerouveal rim thickening on CT and scleral inflammation need not always be scleritic or caused by a pseudotumor. Thick CT sections through the globe may result in volume averaging of the sclerouveal rim and the scleral surface of the globe, which are parallel to the plane of the section. This causes an apparent thickening of the sclerouveal rim in a normal patient. Inflammatory changes after trauma (especially foreign body reactions) and after surgery exhibit similar CT findings.^12^ Orbital infections may also involve the sclera; however, in the vast majority of these cases, other evidence of orbital cellulitis will be demonstrated both clinically and by CT. A careful history is needed when evaluating a globe showing sclerouveal rim thickening on CT.

An earlier research described a case of unilateral orbital myositis of new onset after COVID‐19 without a severe course, in which the patient received topical therapy with a preliminary diagnosis of conjunctivitis, but no improvement was seen.^13^ The medical examination indicated sectoral hyperemia of the temporal area in the bulbar conjunctiva, as well as a significant restriction of right inward gaze. The orbital MRI revealed a widespread fusiform enhancement of the right lateral rectus and superior rectus.^13^ COVID‐19 orbital manifestations might range from severe retro‐orbital discomfort to life‐threatening invasive mucormycosis.^14^ Intubated patients undergoing positive end expiratory pressure ventilation may develop orbital emphysema. Similar with other ocular symptoms, direct viral action, altered immunological state, pro‐inflammatory milieu, and elevated coagulative profile play varying roles in the pathogenesis.^14^ Conjunctival congestion is the most prevalent ocular symptom of severe acute respiratory syndrome coronavirus 2 (SARS‐CoV‐2) infection, involving between 18.4% and 31.6% of individuals with corona virus disease.^14^ Rarely observed in connection with COVID‐19 infection is orbital inflammatory disease. A previous study described a case of orbital pseudotumor after mRNA COVID‐19 immunization in a 40‐year‐old woman who presented with left blepharoptosis for 2 months beginning 1 week after receiving her first Pfizer‐BioNTech mRNA vaccine.^15^ The external examination indicated modest upper eyelid edema and left blepharoptosis. Left lacrimal gland enlargement with homogenous contrast enhancement and diffuse moderate enlargement of the left lateral and superior rectus muscles were detected using orbital magnetic resonance imaging.^15^ This example of orbital pseudotumor formation after vaccination with mRNA vaccine may represent an immune mechanism targeting the orbital tissue.^15^

Oral systemic corticosteroids are the preferred treatment for orbital pseudotumors. A rapid response to steroids is also considered diagnostic. Prednisone doses of 1–1.5 mg/kg per day in children lead to resolution of pain and proptosis within 24–48 h. When the amount of collagenous connective tissue increases, as seen in more chronic forms of orbital pseudotumor, the sensitivity to corticosteroids decreases. Low‐dose radiotherapy can be indicated when corticosteroids fail or are medically contraindicated, or for recurrences when the patient is already receiving corticosteroid therapy.^16^, ^17^, ^18^

## 4 CONCLUSION

Orbital pseudotumor is a rare inflammatory condition that occasionally presents as a chronic condition in children. It is often misdiagnosed as orbital cellulitis or an orbital mass with conjunctivitis. Because this disorder affects vision, it is crucial to diagnose and treat it promptly.

## AUTHOR CONTRIBUTIONS

**Mohammed Almuqbil:** Conceptualization; data curation; formal analysis; funding acquisition; investigation; methodology; project administration; resources; software; supervision; validation; visualization; writing – original draft; writing – review and editing.

## FUNDING INFORMATION

No fund was received for this study.

## CONFLICT OF INTEREST

The author declares no conflict of interest.

## CONSENT

Written informed consent was obtained from the patient's next of kin to publish this report in accordance with the journal's patient consent policy.

## Data Availability

Data sharing is not applicable to this article as no new data were created or analysed in this study.
